# Cardiosphere-derived cells suppress allogeneic lymphocytes by production of PGE2 acting via the EP4 receptor

**DOI:** 10.1038/s41598-018-31569-1

**Published:** 2018-09-06

**Authors:** Luke C. Dutton, Jayesh Dudhia, Brian Catchpole, Hannah Hodgkiss-Geere, Dirk Werling, David J. Connolly

**Affiliations:** 1 Department of Clinical Science and Services, Royal Veterinary College, University of London, Hawkshead Lane, North Mymms, Hatfield, Herts AL9 7TA UK; 2 Department of Pathobiology and Population Sciences, Royal Veterinary College, University of London, Hawkshead Lane, North Mymms, Hatfield, Herts AL9 7TA UK; 3 Institute of Veterinary Science, University of Liverpool, Chester High Road, Neston, CH64 7TE UK

## Abstract

Cardiosphere-derived cells (CDCs) are a cardiac progenitor cell population, which have been shown to possess cardiac regenerative properties and can improve heart function in a variety of cardiac diseases. Studies in large animal models have predominantly focussed on using autologous cells for safety, however allogeneic cell banks would allow for a practical, cost-effective and efficient use in a clinical setting. The aim of this work was to determine the immunomodulatory status of these cells using CDCs and lymphocytes from 5 dogs. CDCs expressed MHC I but not MHC II molecules and in mixed lymphocyte reactions demonstrated a lack of lymphocyte proliferation in response to MHC-mismatched CDCs. Furthermore, MHC-mismatched CDCs suppressed lymphocyte proliferation and activation in response to Concanavalin A. Transwell experiments demonstrated that this was predominantly due to direct cell-cell contact in addition to soluble mediators whereby CDCs produced high levels of PGE_2_ under inflammatory conditions. This led to down-regulation of CD25 expression on lymphocytes via the EP4 receptor. Blocking prostaglandin synthesis restored both, proliferation and activation (measured via CD25 expression) of stimulated lymphocytes. We demonstrated for the first time in a large animal model that CDCs inhibit proliferation in allo-reactive lymphocytes and have potent immunosuppressive activity mediated via PGE_2_.

## Introduction

Cardiac disease is a significant cause of death in humans, accounting for around 25% of all causes of mortality^1^. Recognition that the heart is capable of regeneration^2^, has raised considerable interest over the last decade in identifying possibilities for a cellular therapy for cardiac disease (reviewed in^3,4^). One cardiac progenitor cell type, cardiosphere-derived cells (CDCs), is considered promising for the development of new treatment approaches for cardiac conditions. CDCs are an intrinsic cardiac stem cell population, which have been shown to possess regenerative capabilities^5,6^. A phase 1 clinical trial in humans using autologous CDCs to treat myocardial infarction has demonstrated encouraging results^7,8^. It has been shown in multiple models that CDCs provide beneficial effects to the heart post-injury, with early proposed mechanisms including direct differentiation and contribution to new myocardium^8–10^. However, since the engraftment potential of injected cells is very limited, it is now suggested that paracrine effects confer the majority of the therapeutic outcomes observed^11^. More recently the role of exosomes and micro-RNAs have been identified in the cardioprotective effects seen in CDC therapy^12–15^. The first open-label human study investigating the use CDCs in the treatment of myocardial infarction was limited to using autologous CDCs to avoid subsequent graft-versus-host (GvH) rejection^8^. However, the use of autologous CDCs is time consuming averaging 65 days from tissue biopsy to cell implantation^7^, expensive (due to surgical intervention being required for each individual) and requires cell expansion from diseased myocardium. Thus, the creation of a stem cell ‘master bank’ for off-the-shelf use under allogeneic conditions is an attractive alternative; however, this approach would be complicated by the potential induction of GvH disease^16,17^. Interestingly, mesenchymal stem cells (MSCs) have been shown to possess immunomodulatory properties *in vitro*^18^, through either the secretion of soluble mediators such as transforming growth factor beta (TGF-β1), hepatocyte growth factor (HGF), nitric oxide, prostaglandins and indoleamine 2,3 deoxygenase (IDO)^18^, or direct cell-cell contact via programmed death ligand 1 (PD-L1) and its receptor PD-1^19^.

However, there are conflicting reports on whether the immunomodulatory capacities of MSCs also exist *in vivo*, with studies showing both, cell mediated and humoral responses to transplanted cells^16,20^. In contrast to the findings of these studies using MSCs, allogeneic CDCs have been described as non-immunogenic in a rodent model^21^. Moreover, a study comparing the effect of transplanted MSCs and CDCs in a porcine heart disease model showed the superiority of CDCs in myocardial regeneration^22^.

Since CDCs appear superior to MSCs in animal infarction models, human studies have mainly focussed on treatment post-myocardial infarction, where regeneration of the myocardium is likely to be limited. In non-ischaemic myocardial diseases, such as dilated cardiomyopathy (DCM), where there is an underlying inflammatory component, the use of stem cells as potent immunomodulators may show more promising treatment outcomes^23^. Research utilising CDCs in non-ischaemic myocardial disease has mostly been limited to rodent models^24,25^. Dogs represent a clinically important large animal model for human myocardial disease^26,27^, with non-ischaemic cardiomyopathies being the second most common heart disease seen in canines^28^. Moreover, myocardial disease in dogs share close phenotypic similarities to the equivalent human condition, and this has been particularly well studied in arrhythmogenic ventricular cardiomyopathy (AVC)^29,30^ and DCM in Doberman Pinchers^31,32^. Thus, exploiting naturally occurring non-ischaemic myocardial diseases in dogs, which exhibit close analogy to an equivalent human condition, will act as an essential bridge between discoveries identified in rodent models and achievable clinical therapies. Furthermore, the development of a cellular treatment approach in dogs would have significant translational potential fostering advances in the human field. This is particularly relevant for non-ischaemic DCM where treatment options are limited to implantation of a left ventricular assist device as a bridge to transplantation.

Here, we describe an *in vitro* study examining whether canine CDCs are recognised by allo-reactive lymphocytes from MHC-mismatched donors. Additionally, we investigate mechanisms in this interaction, using this *in vitro* canine model of transplant reactivity.

## Results

### Canine cardiosphere-derived cells express MHC class I, but not MHC class II molecules

**Figure 1:**
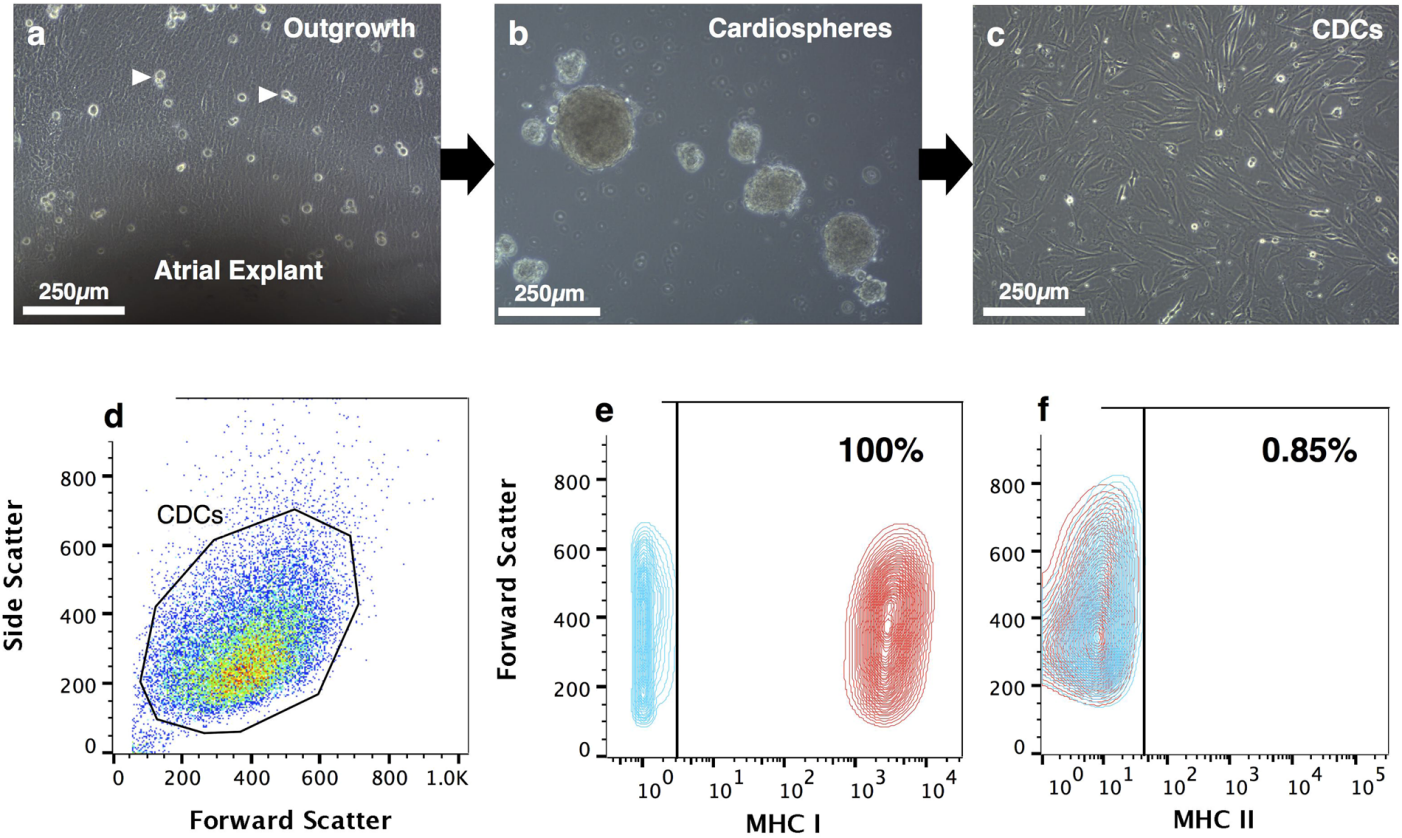
Generation of cardiosphere-derived cells (CDCs) and MHC class I and class II phenotype. Atrial explants were first plated onto fibronectin-coated plastic, which allowed outgrowth cells to develop, over which phase-bright cells proliferate (**a**). Cells were harvested and plated onto a low attachment surface to generate cardiospheres (**b**). Cardiospheres are then re-attached to tissue culture plastic to form adherent monolayer CDCs (**c**). Flow cytometry analysis shows gated CDCs (**d**) with a high expression of MHC class I molecules (**e**) and very low expression of MHC class II molecules (**f**). Blue contours denote isotype control and red contours denote antibody labelled samples. Scale bars = 250 µm.

A layer of stromal like cells emerged from the atrial explants over which phase-bright cells proliferated (Fig. 1a). These cells formed spheres when plated on a low attachment surface (Fig. 1b), which were able to grow as a monolayer when re-plated on fibronectin-coated plastic to form CDCs (Fig. 1c). Cells generated by this technique were recently described by us to express surface antigens with different intensity, and were phenotyped as CD105^++^, CD90^+^, c-Kit^−^ and CD45^−^ ^33^. Flow cytometry analysis showed that all CDCs expressed MHC I molecules (99.7 ± 0.09%, MFI value 2707.67 ± 370.30, Fig. 1e), with few cells expressing MHC class II (1.17 ± 0.59%, MFI value 6.37 ± 0.90, Fig. 1f). To ensure full MHC-mismatching for subsequent experiments, we genotyped DLA-88 (encoding MHC I) and DLA-DRB1 (encoding MHC II) of all dogs involved in ths study (Table 1). Only one shared allele between donor animals D2 and D5 was found.

**Table 1:** Donor characteristics and MHC genotypes of animals used in this study.

	Breed	Age (years)	Sex	Reason for euthanasia	DLA-88 genotype	DLA-DRB1 genotype
D1	Irish Wolfhound	0.5	Male (entire)	Pneumonia	01201/50101	00101/01501
D2	Labrador	6	Male (neutered)	Myelopathy	00402/50801	00601/01201
D3	Cross-breed	5	Male (neutered)	Myelopathy	04401/04501	00102/01701
D4	Dachshund	5	Male (neutered)	IVDD	00601/02101	00203/07301
D5	American Cocker Spaniel	4	Female (entire)	IVDD	00402/00402	00601/00601

### Co-culture of allogeneic CDCs with lymphocytes does not increase cell death, but completely inhibits their proliferation and reduces CD25 expression

**Figure 2:**
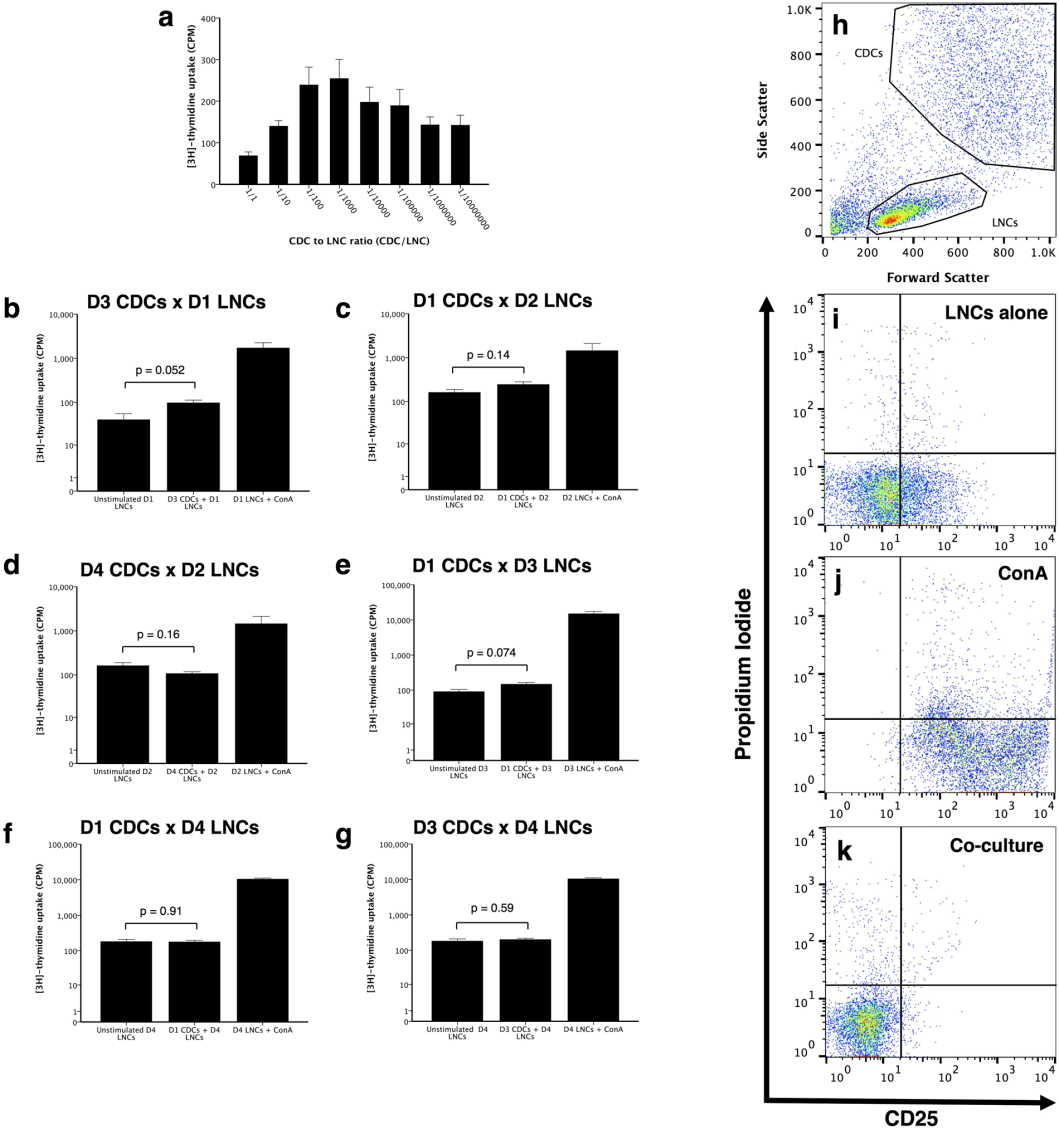
Mixed lymphocyte reactions and flow cytometry analysis of CD25 expression on lymph node cells (LNCs) in response to allogeneic donor cardiosphere-derived cells (CDCs). Initial titration experiments revealed a peak stimulation of LNCs at between 1:100 and 1:1000 ratios of CDCs:LNCs (n = 3, with triplicate samples in each **a**). Different donor CDCs cultured with LNCs, at a ratio of 1:500 CDCs:LNCs. At this ratio, there was no difference between resting LNC proliferation and those cultured with allogeneic CDCs (n = 4 dogs, with triplicate samples for each, p > 0.05 **b**–**g**). FACS analysis of co-cultured cells allowed LNCs to be gated **h**. Graphs **i**–**k** show only gated LNCs with simultaneous CD25 and PI staining analysed for both LNC activation and death respectively. ConA treated LNCs had more CD25 signal compared to LNCs alone without stimulation (n = 4, p < 0.001, **i** and **j**). Co-culture LNCs with CDCs had less CD25 levels than LNCs alone or LNCs stimulated with ConA (n = 3, p < 0.001, **k**). Bars represent mean ± SEM of 3 different dogs for **a**, and mean ± SEM of triplicate results in each **b**–**g**.

To assess whether the high level of MHC I expression would support a GvH response, we next explored the response of lymph node cells (LNCs) to MHC-mismatched allogeneic CDCs. Initial titration experiments using allogeneic CDC in different ratios with LNCs revealed low [^3^H]-thymidine uptake in mixed lymphocyte reactions (MLRs), with a peak response at a CDC:LNC ratio of 1:100–1:1000 (Fig. 2a). For technical reasons, we therefore used a ratio of 1:500 in subsequent assays. Further co-culture experiments confirmed a lack of LNC proliferation in response to allogeneic CDCs (p > 0.05, compared to LNC only controls, Fig. 2b–g). Different biological replicates exhibited substantially varied p-values (e.g. p = 0.052 versus 0.91), which likely reflect the inherent individual variation between dogs of different breeds. To assess whether this lack of response was due to cell death in LNCs or CDCs, we assessed the degree of cell death in co-cultures using propidium iodide (PI) staining. Interestingly, there was no difference in the number of either PI^+ve^ CDCs or LNCs under any co-culture condition, compared to each cell type cultured alone (PI^+ve^ LNCs p = 0.185. PI^+ve^ CDCs p = 0.409, Fig. 2i–k).

To investigate whether co-culture of CDCs and LNCs only impacted on proliferative responses, and to confirm the [^3^H]-thymidine results, allogeneic LNCs were assessed for the IL-2 receptor (CD25) expression as a marker for early lymphocyte activation, with the gating strategy shown in Supplementary Fig. S1. In unstimulated LNCs, a low percentage as well as a low intensity for CD25 staining was seen (n = 3, 16.54 ± 0.19% and MFI = 30.72 ± 1.16, Fig. 2i). The low CD25 staining was not due to an inherent incapability of LNCs to express this surface antigen, as ConA-stimulated the number as well as the intensity of LNCs expressing CD25 (n = 4, 95.87 ± 0.45% and MFI = 1193.29 ± 63.94, Fig. 2j). When MHC-mismatched CDCs were cultured with lymphocytes, the resulting CD25 staining intensity and percentage positive LNCs was less compared to LNCs cultured alone (n = 3, 2.81 ± 0.41% and MFI = 6.34 ± 0.14, p < 0.001, Fig. 2k). This reduction was not due to the induction of apoptosis, as additional Annexin V staining indicated that LNCs did not induce apoptosis in CDCs, as the percentage of Annexin V positive cells showed now significant difference when CDCs were cultured with or without LNCs (p = 0.81, Fig. S2).

### CDCs exhibit a dose-dependent inhibition of lymphocyte proliferation

**Figure 3:**
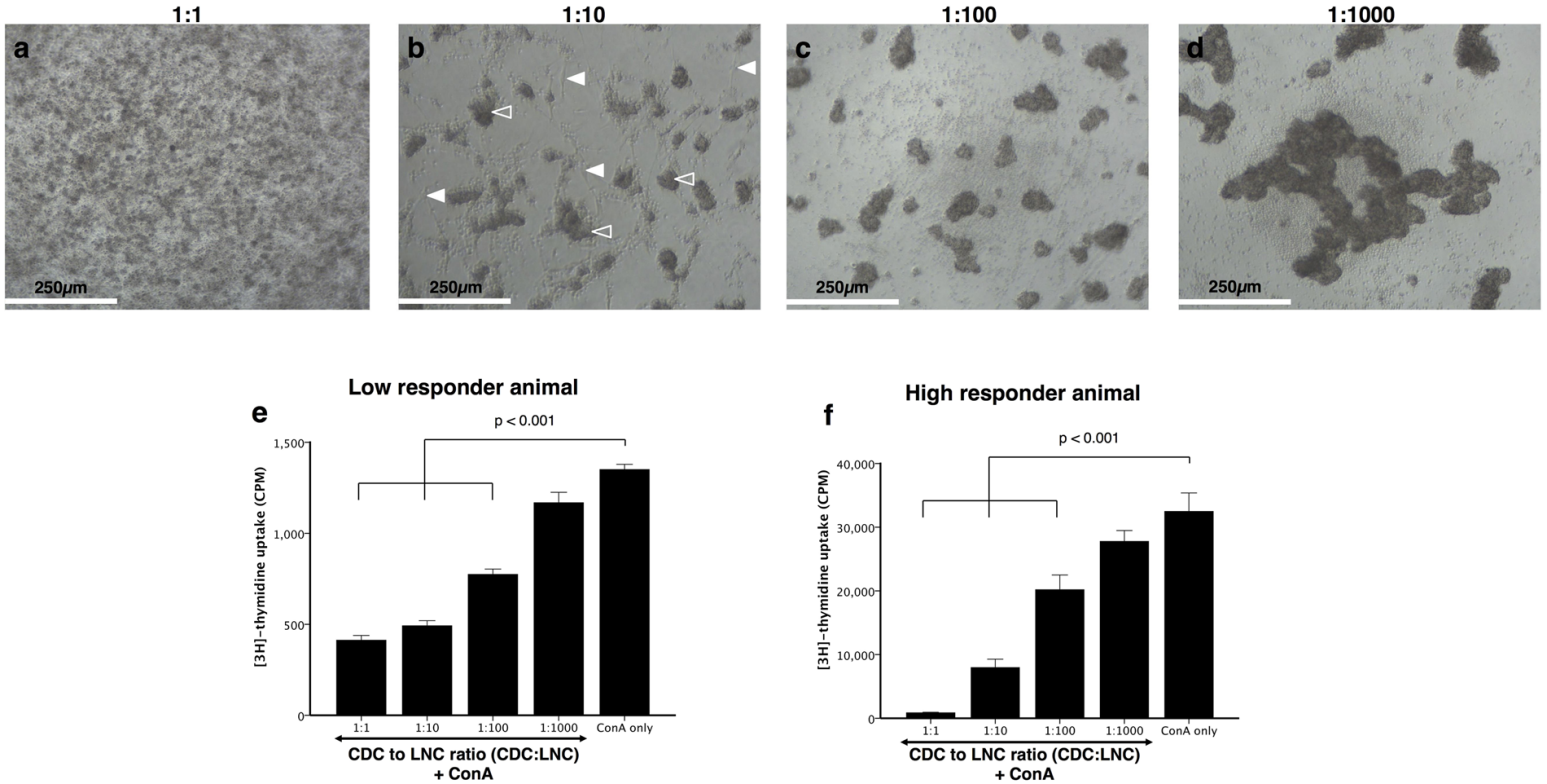
Dose-response relationship in cardiosphere-derived cell (CDC) suppression of lymph node cell (LNC) activation. All cultures contained ConA, with ratios of 1:1 to 1:1000 of CDC:LNCs. Panels **a–d** are representative photomicrographs of dose-response experiments, showing little proliferation at a 1:1 ratio (**a**) and increasing lymphocyte blast formation with fewer CDCs (**b**–**d**). Filled arrows indicate the CDCs and open arrows the LNC blast formation. There was inhibition of LNC activation at 1:1, 1:10 and 1:100 ratios (n = 3, p < 0.001, **e** and **f**), with inhibition lost at 1:1000 (n = 3, p = 0.298). The response is similar in a low responder animal (**e**) and a high responder animal (**f**), the third biological repeat is not displayed. Bars represent mean ± SEM of triplicate sample, n = 3 dogs; Scale bars = 250 µm.

Having established that CDCs down-regulate baseline CD25 expression in LNCs, we next investigated whether CDCs were also capable of inhibiting ConA-activated LNC functionality. Figure 3a–d shows photomicrographs of the suppressive reaction at different CDC concentrations. At a high number of CDCs, there was little to no lymphocyte blast formation in response to ConA stimulation (Fig. 3a), whereas in the presence of a low number of CDCs, LNCs were able to respond to ConA to form lymphocyte blasts (Fig. 3d). When CDCs and LNCs were cultured at a 1:1 ratio, the ConA response was reduced by 69.42 ± 1.92% for a low, and 97.36 ± 0.28% for a high responding animal (p < 0.001, Fig. 3e and f). This inhibition exhibited a dose-response pattern with decreasing numbers of CDCs, and there was no significant difference in inhibition at a ratio of 1:1000 (p > 0.05). The inhibition was MHC-unrestricted and was similar for both low- and high responder animals.

**Figure 4:**
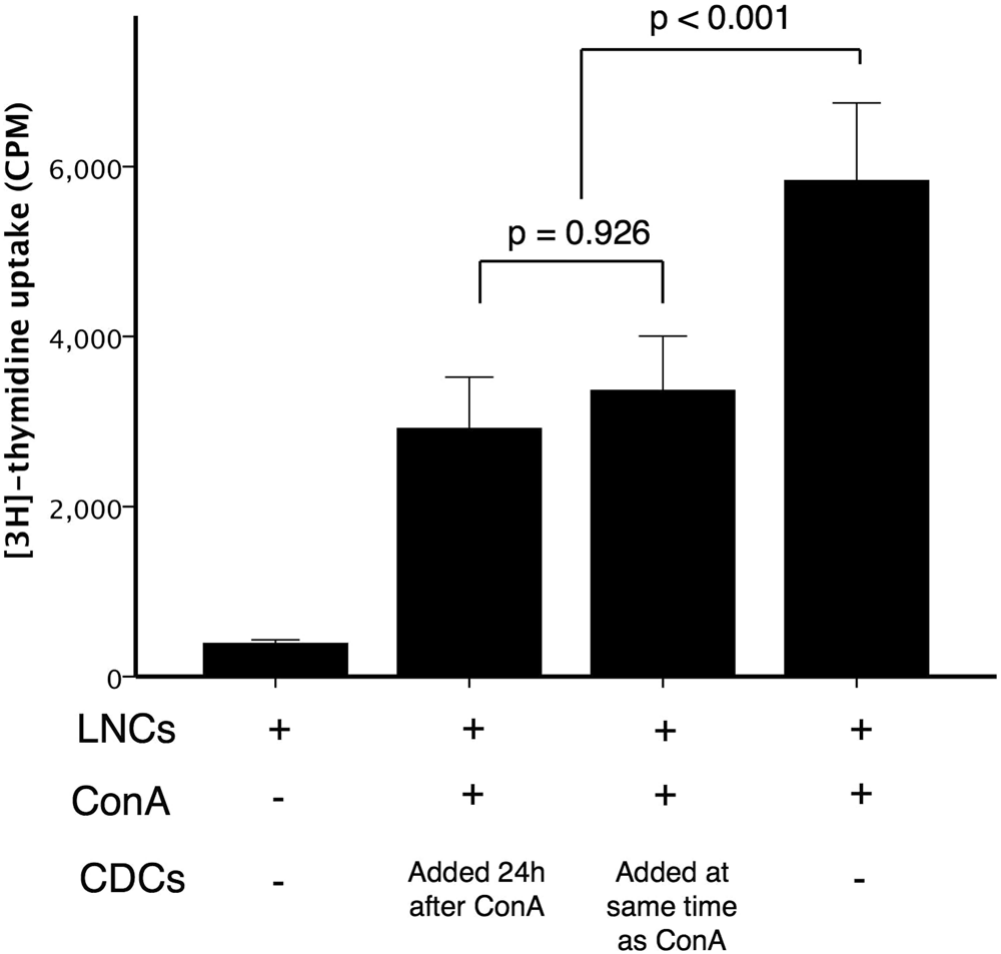
Inhibition of pre-activated lymphocytes by cardiosphere-derived cells. Lymph node cells (LNCs) were culture alone or with ConA. In one setup cardiosphere-derived cells (CDCs) were added at the same time as ConA, in another the CDCs were added 24 h after LNCs had been exposed to ConA. The LNC:CDC ratio was 10:1. Inhibition of LNC activation by CDCs was independent of the preactivated status of the lymphocytes (Bars represent mean ± SEM, n = 3 dogs, with triplicate samples in each, p = 0.926).

As this inhibition of the proliferative response was with naive LNCs, we next investigated responses of pre-activated lymphocytes to assess whether pre-activation could rescue the lymphocytes from CDC induced anergy. As shown in Fig. 4, regardless of the previous activation status of the LNCs, proliferation was inhibited by the addition of CDCs (p < 0.001).

### CDCs inhibit activation of naive lymphocytes

**Figure 5:**
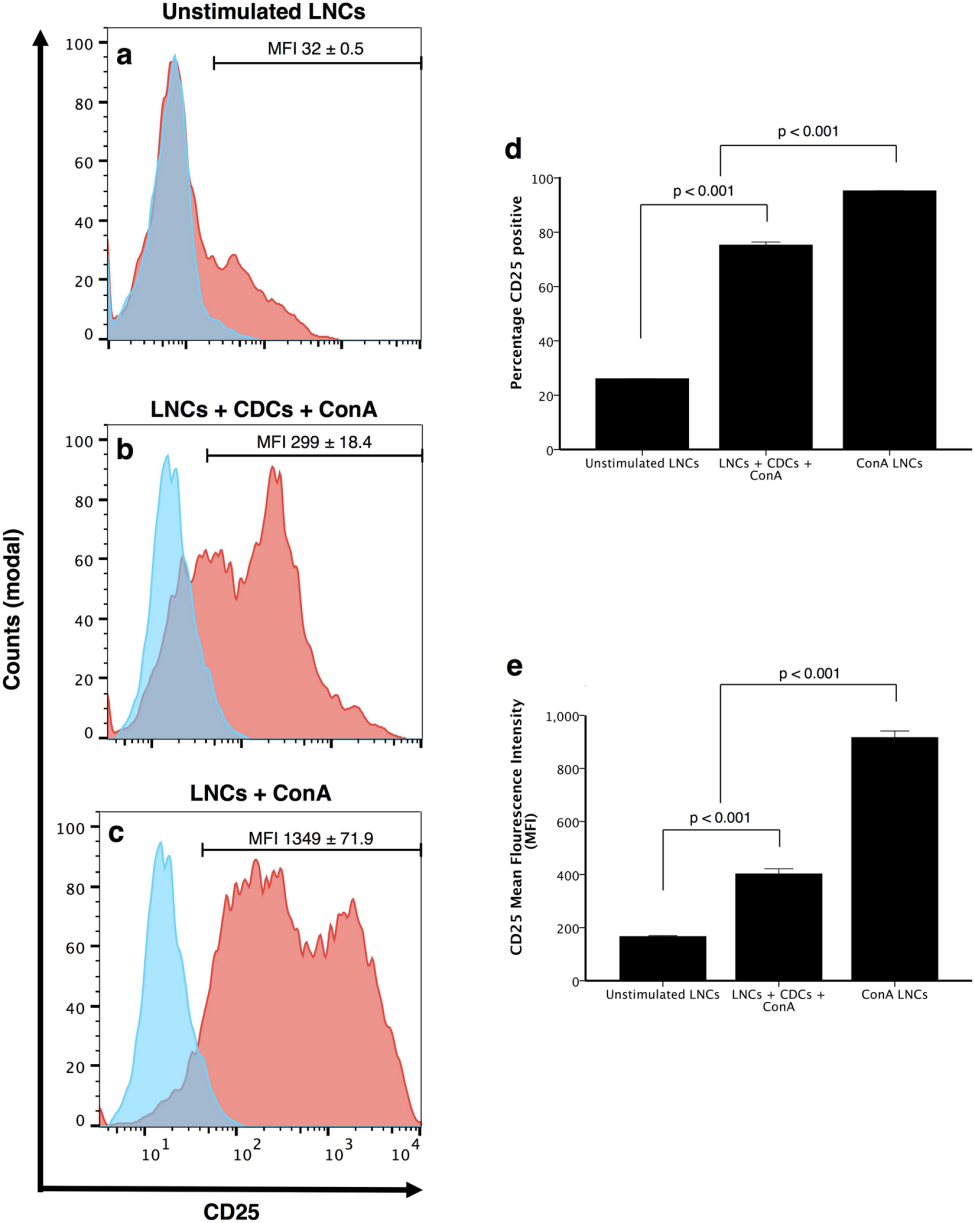
Cardiosphere-derived cells (CDCs) inhibit lymph node cell (LNC) activation in the presence of a mitogen. Flow cytometry analysis shows gated LNCs only in **a–c**. LNCs have low CD25 staining intensity when unstimulated (**a**) more CD25 staining when cultured with CDCs and stimulated with ConA (**b**) and the most CD25 level when stimulated with ConA without CDCs (**c**). Both the percentage of CD25^+^ cells and the CD25 staining intensity are less in co-culture conditions compared to ConA alone (% CD25^+^ reduction = 28.30 ± 4.72%, n = 3, p < 0.001 **d**). MFI ConA = 1193.29 ± 62.94, MFI co-culture = 319.44 ± 40.23, n = 4, p < 0.001 **e**). Bars represent mean ± SEM.

We next sought to assess whether CDCs down-regulate activation of LNCs by CD25 expression in co-cultures. Unstimulated LNCs exhibit low CD25 staining intensity and a low number of positive cells (Fig. 5a). When CDCs are co-cultured with ConA-stimulated LNCs, fewer CD25 positive cells were detected compared to ConA-stimulated LNCs without CDCs (p < 0.001, Fig. 5b–d), with the staining intensity for CD25 on LNCs also being less (MFI ConA = 1193.29 ± 62.94, MFI co-culture = 319.44 ± 40.23, p < 0.001, Fig. 5e).

### Inhibition of lymphocyte activation and proliferation is partially mediated by soluble factors

**Figure 6:**
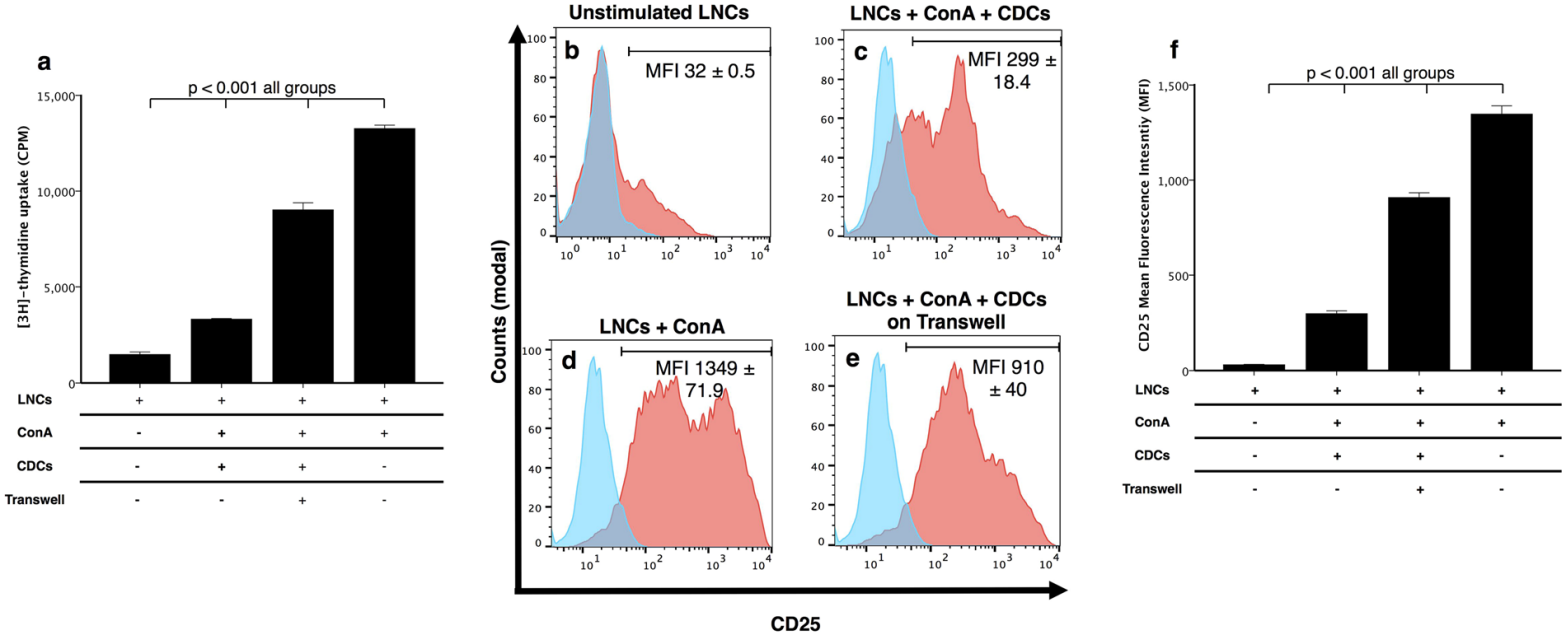
Suppressive activity of cardiosphere-derived cells (CDCs) is partially mediated by soluble factors. [^3^H]-thymidine uptake shows that the separation of stimulated lymph node cells (LNCs) from the CDCs using a transwell partially restores the proliferation of the LNCs compared to when LNCs are in direct contact with CDCs (p < 0.001, **a**). Flow cytometry analysis shows when lymph node cells (LNCs) are cultured alone they express low CD25 (**b**) more CD25 when stimulated with ConA and cultured with CDCs (**c**) and the highest expression when only stimulated with ConA **d**). The use of a transwell to separate the CDCs and LNCs results in more LNCs expressing CD25 than when CDCs and LNCs are in direct contact (**e**). Histograms **b**–**e** show CD25 expression (red histogram) relative to isotype control (blue histogram). CD25 mean fluorescence intensity (MFI) shows all groups differ significantly (p < 0.001, **f**). Data is representative of 3 dogs; each experiment is run in triplicate. Bars represent mean ± SEM.

As inhibition of proliferation may require direct cell contact, soluble factors, or both, we next assessed whether the inhibition of lymphocyte activation indeed required the physical interaction of both cell types. Interestingly, when CDCs were separated from the stimulated LNCs in a transwell culture system, proliferation of the LNCs was 43.04 ± 2.63% more than for those LNCs cultured in direct contact with CDCs (p = <0.001, Fig. 6a). The same pattern was observed for CD25 expression, with the staining intensity for CD25 on LNCs separated from CDCs using a transwell being more than when LNCs are in direct contact with CDCs (n = 3, direct contact: 69.30 ± 0.80%, MFI 299.00 ± 13.00, transwell: 88.70 ± 0.47%, MFI 910 ± 23.09, p < 0.001, Fig. 6b–f). Since these data suggested that both, soluble mediates and direct cell contact is required for LNC inhibition by CDCs, we explored possible mechanisms of this interaction.

### Prostaglandin E_2_ down-regulates CD25 expression on LNCs via the EP4 receptor

Prostaglandins have been described to show strong immunosuppressive functions, and are secreted by a variety of stem cells^34–36^.

**Figure 7:**
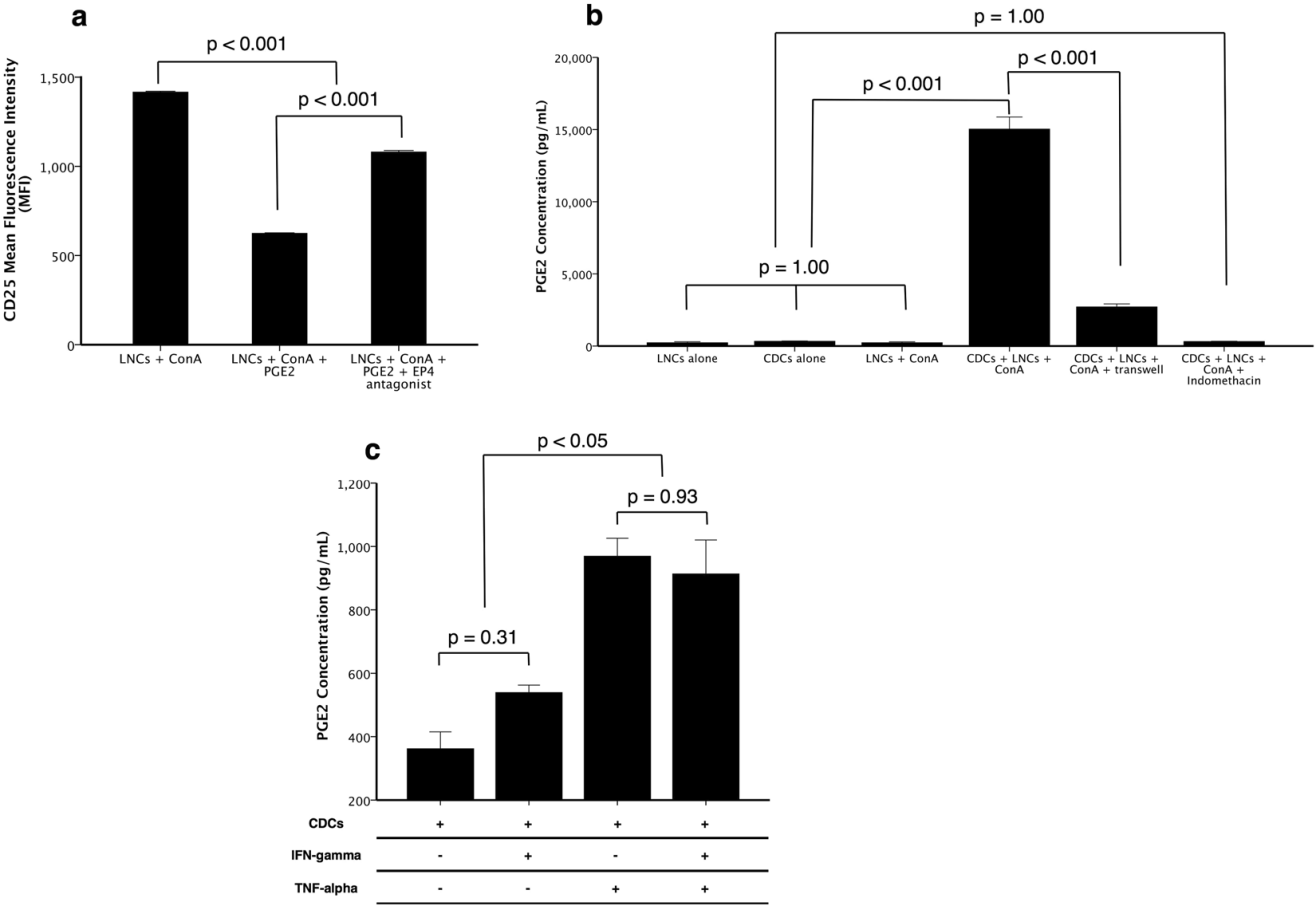
Prostaglandin E_2_ (PGE_2_) inhibits CD25 expression on lymph node cells (LNCs) via the EP4 receptor and is produced by cardiosphere-derived cells (CDCs) in response to direct contact with LNCs and TNF-α. When LNCs are activated using ConA, CD25 has high staining intensity, which is significantly less when they are treated with PGE_2_ (n = 3, p < 0.001, **a**). This effect is partially blocked by the addition of L-161982, a specific EP4 receptor antagonist (n = 3, p < 0.001, **a**). Additionally, when CDCs are cultured with stimulated LNCs, they produce high levels of PGE_2_ compared to either cell type alone (n = 3, p < 0.001, **b**). PGE_2_ production is ameliorated when CDCs are separated from LNCs by a transwell (n = 3, p < 0.001, **b**). Indomethacin completely prevents CDCs from producing PGE_2_ (n = 3, p = 1.00, **b**). PGE_2_ production could be induced in CDCs cultured with 50 ng mL^−1^ TNF-alpha (n = 3, p < 0.05, **c**) but not with IFN-gamma (n = 3, p = 0.31, **c**). Bars represent mean ± SEM of 3 dogs.

Since our data indicated that a key mechanism in the potential induction of anergy in lymphocytes is the CDC-induced down-regulation of CD25, we explored the potential involvement of the prostaglandin pathway. ConA-activated LNCs treated with PGE_2_ had less CD25 staining intensity than when left untreated (p < 0.001, Fig. 7a), and this effect was abrogated by the addition of L-161982, a highly selective EP4 antagonist (p < 0.001, Fig. 7a). ELISA analysis of MLR supernatants showed that CDCs produced low amounts of PGE_2_ when cultured alone (332 ± 21 pg mL^−1^, Fig. 7b). However, when cultured with ConA-activated LNCs, the PGE_2_ concentration was approximately 45-fold more (15,035 ± 835 pg mL^−1^, Fig. 7b). Interestingly, PGE_2_ concentration was less when a transwell was used to separate the two cell types versus when direct contact is allowed, although it was still more than baseline LNC or CDC levels (2713 ± 204 pg mL^−1^, p < 0.001, Fig. 7b). Indomethacin, a selective cyclooxygenase (COX) inhibitor, reduced PGE_2_ production by CDCs to baseline levels (p = 1.00, Fig. 7b).

### PGE_2_ is produced by CDCs in response to TNF-α but not IFN-γ

To explore the involvement of IFN-γ and TNF-α in stimulating PGE_2_ synthesis, CDCs were cultured in the presence or absence of IFN-γ, TNF-α or both, and PGE_2_ production measured after 48 h by ELISA. IFN-γ did not stimulate PGE_2_ synthesis in CDCs (p = 0.31) whereas TNF-α was moderately stimulatory (p < 0.05, 969.2 ± 23.4 pg mL^−1^, Fig. 7c). The combination of the two cytokines did not increase PGE_2_ production further (p = 0.93, Fig. 7c).

### Inhibition of PGE_2_ synthesis ameliorates CDC suppressive function

**Figure 8:**
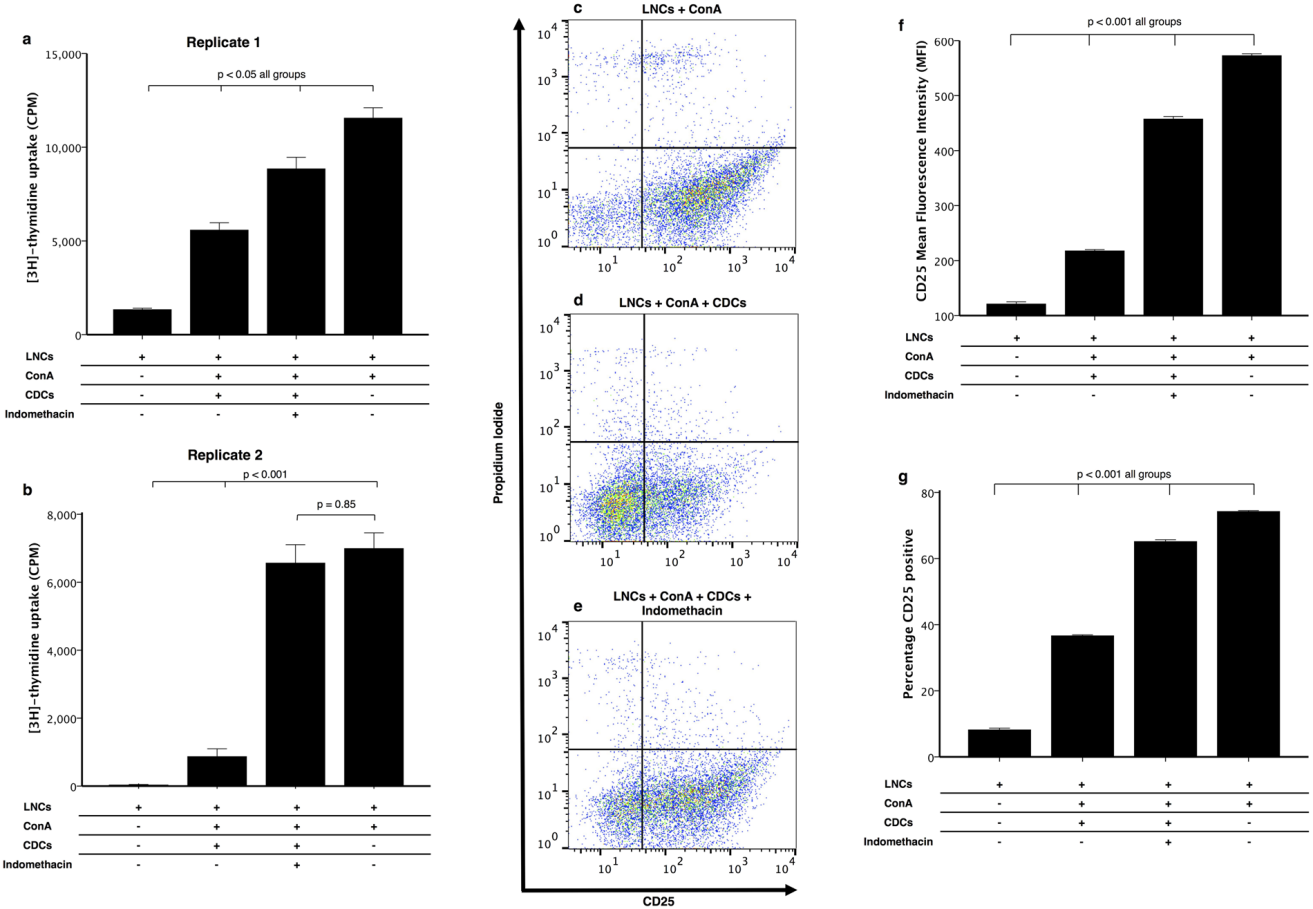
Inhibition of prostaglandin synthesis restores both lymphocyte proliferation and activation. Cardiosphere-derived cells (CDCs) were co-cultured with lymph node cells (LNCs) in the presence of ConA at a ratio of 1:10 (CDC:LNC). The addition of indomethacin (20 µM), a cyclooxygenase inhibitor, significantly increased the proliferation of LNCs; independent of donor and recipient cell line (n = 3, p < 0.05, **a** and **b**). **a** and **b** are biological replicates with technical triplicates in each, bars represent mean ± SEM of the technical replicates. CD25 signal intensity was also more when indomethacin was added to co-culture of CDCs and LNCs in the presence of ConA compared to when no indomethacin is added (n = 3, p < 0.001, **c**–**f**). The percentage of cells expressing CD25 was also partially restored by the addition of indomethacin (n = 3, p < 0.001, **g**). Bars represent mean ± SEM of 3 dogs.

To confirm the role of PGE_2_ in inducing lymphocyte anergy, we conducted experiments using indomethacin, since we confirmed that this reduces PGE_2_ synthesis to baseline in CDCs. The proliferation of ConA-stimulated LNCs in co-culture with CDCs is more when indomethacin is added (p < 0.001, Fig. 8a and b). In one biological replicate, the proliferation was the same control levels (p = 0.85 compared to ConA stimulated cultures, Fig. 8b). Additionally, there were more CD25^+^ lymphocytes and more CD25 staining intensity, supporting the effects seen on proliferation (p < 0.001, Fig. 8c–g).

### CDC secretion of TGF-β1 is not affected by the presence of activated LNCs

**Figure 9:**
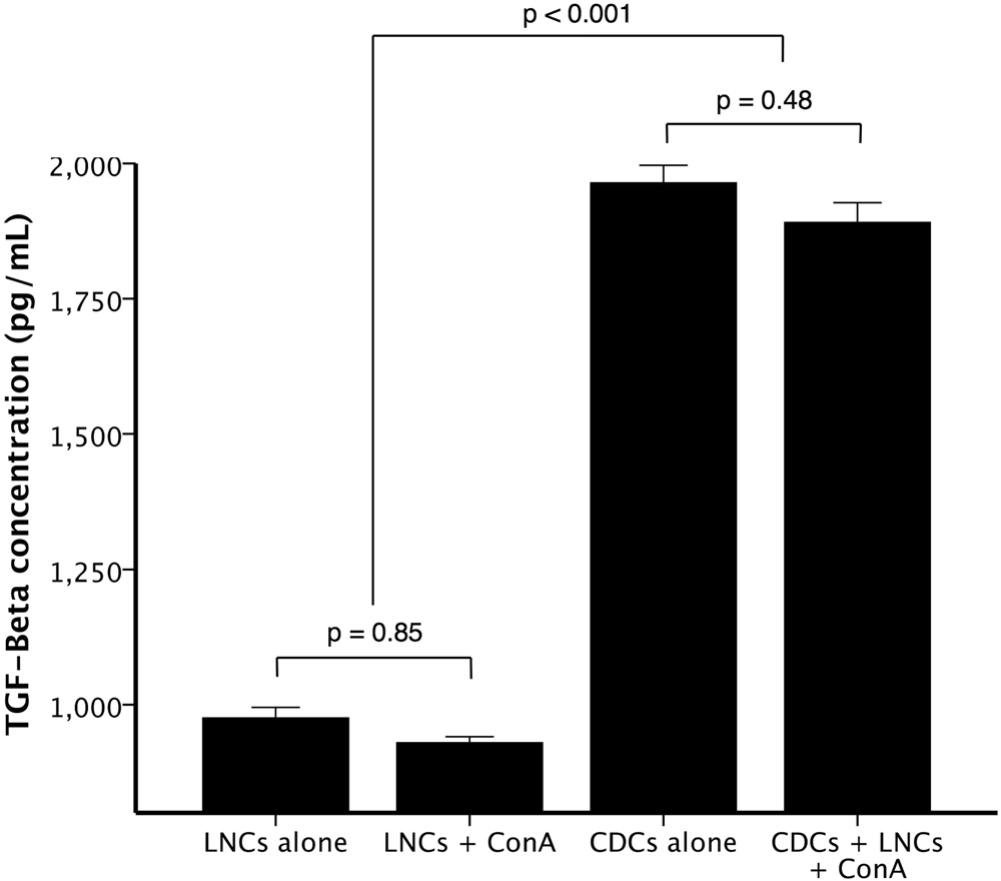
TGF-β is secreted by cardiosphere-derived cells (CDCs) at a basal level, and this secretion is unaffected by the presence of activated lymphocytes. Unstimulated LNCs and ConA-stimulated LNCs produced similar low TGF-β concentrations (n = 3, p = 0.85). CDCs produced more TGF-β than LNCs alone (n = 3, p < 0.001), but this basal secretion was unaffected by the addition of Con-A stimulated LNCs to the CDCs (n = 3, p = 0.48). Bars represent mean ± SEM of 3 dogs.

Other stem cell types secrete the potent immunosuppressive cytokine TGF-β1^37,38^. We also tested whether CDCs secrete TGF-β1, which may further enhance the reduction in LNCs proliferation and CD25 expression^39,40^. We analysed supernatants from CDCs cultured alone, and found that when comparing the same cell numbers, the TGF-β1 concentration was not different between CDCs cultured alone and CDCs cultured with activated LNCs (n = 3, 1963.9 ± 32.6 versus 1891.0 ± 36.5, p = 0.48, Fig. 9). Culture supernatants taken from the MLR described above contained higher levels of TGF-β1 when LNCs were cultured with CDCs, compared to LNCs alone (p < 0.001, Fig. 9). This indicates that CDCs produce TGF-β1 at a basal level, which is not affected by the presence of activated LNCs.

## Discussion

In the present study, we have demonstrated that canine CDCs induce a state of anergy in allogeneic lymphocytes, and this can be mainly attributed to secretion of prostaglandin (PGE_2_) acting via the EP4 receptor on responding lymphocytes. Based on the data obtained, we hypothesise that the mechanism through which allogeneic CDCs evade immune recognition shares a common pathway by which they exhibit their protective cardiac effects. The proposed mechanism is outlined in Supplementary Fig. S3 and expanded below. Therefore our study provides the first evidence for how CDCs evade graft-versus-host rejection, which supports their use for allogeneic transplantation in canine patients. Furthermore, we propose that the immunomodulatory properties of CDCs described here may have important therapeutic consequences in non-ischaemic cardiomyopathies where inflammation is a key component of the underlying pathogenesis and a mechanism for driving disease progression^41,42^. Indeed, CDCs have been reported to attenuate scar formation and fibrosis in the heart post-injury via release of as yet unidentified paracrine factors^11^. In addition, canine DCM and AVC represent a clinically relevant model for human non-ischaemic myocardial disease with swift translational potential.

Cell-donor animals used in the present study expressed mostly different genotypes for DLA-88 and DLA-DRB1, which encode the most polymorphic regions of MHC class I and class II molecules^43,44^. This allowed true allogeneic MHC-mismatched co-culture of CDCs and LNCs. Canine CDCs expressed MHC class I, but not class II molecules, which is consistent with other adult stem cell types^45^. MHC I expression should allow direct allo-recognition by T-cells^46,47^. Thus, while CDCs generated within the present study might have been expected to induce an allogeneic response, it was therefore noteworthy that this did not occur. In addition to the absence of a proliferative response, we also did not detect an increase in CD25 expression by LNCs in response to allogeneic CDCs. CD25 (the α-chain of the IL-2 receptor) is an early marker of T-cell activation, whose up regulation in response to mitogenic and cellular stimuli leads to IL-2 secretion by T-cells^48^. Even more interesting was the subsequent observation that co-culture of CDCs with allogeneic LNCs resulted in a decrease in CD25 baseline expression by LNCs which could not be attributed to increased cell death through bulk effect or induction of apoptosis/necrosis. Importantly, the absence of increased CD25 expression corroborates our proliferation data, suggesting that allogeneic CDCs both prevent early activation as well as effector function of LNCs. As CD25 acts as a positive feedback autocrine loop for IL-2 driven clonal expansion of T-cells^49^, our observation would suggest that CDCs act by preventing lymphocyte activation. A proposed mechanism for this by MSCs involves the production of matrix metalloproteinases (MMPs) by stem cells and subsequent MMP-mediated cleavage of the IL-2 receptor on lymphocytes to inhibit their proliferative ability^50^. We speculate that a similar mechanism of lymphocyte inactivation is elicited by CDCs as our observation regarding the lack of immunogenicity in canine CDCs fits with observations in other adult stem cells which have been shown in a wide variety of small and large animal models to not elicit allogeneic lymphocyte responses^18,49,51^. However, it is possible that if CDCs were injected intracardially, the microenvironment of the heart may cause MHC II upregulation and potential immune recognition^21,52^. Therefore, this would require further investigation using an *in vivo* canine model.

Independent of this, our finding that CDCs can induce a state of anergy in allogeneic lymphocytes is important in the clinical context. This conclusion is based on the existing literature, where as in human cardiac disease^42,53,54^; canine non-ischaemic myocardial diseases frequently involve a multifocal inflammatory infiltrate of T-lymphocytes, associated with cardiac fibrosis^30,55^ especially in cases of sudden cardiac death. Furthermore, a number of key pro-inflammatory cytokines have been shown to be elevated in canine non-ischaemic cardiomyopathy^56^. However a limitation of our study is that the activation status (CD25) of infiltrating lymphocytes in canine myocardial disease is at present unknown, and therefore it would be important to ascertain if DCM hearts of dogs contain activated lymphocytes to maximise the therapeutic potential.

In order to further elucidate the mechanisms driving CDC-dependent suppression of LNCs, we showed that this effect was dependent to a large extent on direct cell-cell contact, as co-culture of both cell types in a transwell system almost completely restored both proliferation and CD25 expression in stimulated LNCs. However, as restoration was not complete, soluble mediators may additionally contribute either by supporting the effect mediated through cell-cell contact or by direct contact enhanced soluble factor release. In MSCs, a direct contact mechanism of lymphocyte inhibition involving interaction of programmed death ligand 1 (PD-L1) and its receptor (PD-1) on lymphocytes has been implicated^19,57^. This pathway has also been shown to inhibit lymphocyte proliferation in human c-Kit^+^ cardiac stem cells^52^. In addition, secreted factors such as TGF-β1^37^, hepatocyte growth factor (HGF)^58^, MMPs, indoleamine 2,3-dioxygnase (IDO)^59^, prostaglandin E_2_ (PGE_2_)^60^ and nitric oxide (NO)^61^ are known to further inhibit T-cell responses in MSC co-culture systems.

Indeed, we found that TGF-β1 was increased in co-cultures with CDCs and LNCs, which may inhibit lymphocyte proliferation^62^. Whilst TGF-β1 may provide a pro-fibrotic signal^63^, current *in vivo* evidence is consistent in showing that CDCs are anti-fibrotic, in mouse, rat, porcine, canine and human models^7,24,64–66^. Therefore it is unlikely that the TGF-β1 released from the CDCs would be a strong pro-fibrotic signal. Additionally prostaglandins, specifically PGE_2_, have been demonstrated to suppress CD25 expression on bovine lymphocytes via the prostaglandin receptor, EP4^35^. Binding of PGE_2_ to the EP4 receptor on lymphocytes causes increased cAMP, which activates protein kinase A pathways leading to modulation of NF-AT and AP-1. This leads to reduced binding of these transcription factors to the promoter regions of IL-2 and the IL-2 receptor (encoding CD25), causing significant reduction in the production of IL-2 and it’s receptor^67^. Here, we demonstrated that CDCs produce PGE_2_ in high concentrations when exposed to ConA-activated lymphocytes, and that inhibition of PGE_2_ production restores lymphocyte activation and proliferation. Interestingly, direct cell-cell contact was necessary for the higher secretion of PGE_2_, which correlated with the rescue of lymphocyte CD25 expression. Thus, PGE_2_ release by CDCs through direct contact is the primary stimulus for the immunosuppression observed. We were able to induce PGE_2_ secretion by canine CDCs following stimulation by the pro-inflammatory cytokine TNF-α, which is also a feature observed in other stem cells^68,69^. However, this effect was only moderate in comparison to the levels of PGE_2_ obtained in direct co-culture, again indicating the involvement of separate mechanisms. Therefore, it is possible that direct contact either enhances the release of cytokines or induces a more complex bidirectional signalling.

It is worth mentioning that this immune-modulation via PGE_2_ could potentially facilitate allogeneic applications in a clinical setting, but may also have direct reparative effects on the myocardium. Compelling evidence suggests that PGE_2_, also acting via the EP4 receptor in the heart, results in the development of a protective effect against inflammation^70^ as well as cardiomyocyte replacement^71^.

In conclusion, we demonstrate for the first time that CDCs induce a potential state of anergy in allogeneic lymphocytes, and propose a mechanism for this effect through the secretion of PGE_2_ by CDCs, acting via the EP4 receptor. The PGE_2_ produced by CDCs may also have a dual function in facilitating cardiac repair, possibly attenuating diverse inflammatory pathways, which act as a key pathological substrate in non-ischaemic myocardial disease. Our findings support the potential for translation to *in vivo* studies in dogs to advance the clinical use of allogeneic CDCs in regenerative cardiac therapy, particularly for the management of non-ischaemic cardiomyopathy for which currently there are limited treatment options in the canine species.

## Methods

### Study animals

Hearts and popliteal lymph nodes were removed from five dogs at post-mortem with owners’ informed consent, as approved by the Ethics and Welfare Committee (Approval number: URN 2013 1246) at the Royal Veterinary College. All experiments were performed in accordance with the relevant guidelines and regulations. The donor characteristics are summarised in Table 1.

### Preparation of cardiosphere-derived cells

Hearts were aseptically removed from cadavers, and atrial tissue dissected and transported in chilled cardiac explant medium (CEM) containing Iscove’s Modified Dulbecco’s Medium (IMDM), 20% fetal bovine serum (FBS), 1% L-glutamine, 1% penicillin-streptomycin (P/S) (all Thermo Fisher Scientific) and 0.1 mmol L^−1^ 2-mercaptoethanol (2-ME) (Sigma-Aldrich). Cardiosphere-derived cells (CDCs) were prepared as described elsewhere^33,64,65^. Briefly, atrial tissue was diced finely to 0.1 mm^³^ and digested in 0.2% trypsin and 0.1% collagenase IV (both from Thermo Fisher Scientific) three times for 5 min each at 37 °C. The tissue was washed with Dulbecco’s phosphate-buffered saline (DPBS) between each digestion. Explants were transferred onto fibronectin (Thermo Fisher Scientific) coated 25 cm^2^ tissue culture flasks (Greiner Bio One) in CEM media and incubated in standard tissue culture conditions (37 °C and 5% CO_2_ in humidified air) to allow phase-bright cells to proliferate over a stromal-like cell monolayer. These cells were harvested with TrypLE Express (Thermo Fisher Scientific) and seeded at a density of 1 × 10^5^ cm^−2^ on Ultra-Low Attachment flasks (ULA; Corning). The phase-bright cardiospheres were collected and seeded onto fresh fibronectin-coated flasks to yield a monolayer of CDCs. Cells were passaged when 60–80% confluent using 0.25% trypsin (Thermo Fisher Scientific) and re-seeded at 6 × 10^3^ cm^−2^. CDCs were cryopreserved in CellBanker 2 medium (AMS Biotechnology Ltd.) at a density of 1 × 10^6^ mL^−1^ at −80 °C for 24 h before transfer to liquid nitrogen storage. CDCs were characterised as described in our previous work^33^.

### Isolation of lymph node cells

Popliteal lymph nodes were removed from the dogs and placed in lymphocyte medium (LM) consisting of RPMI 1640 (Sigma Aldrich), 10% FBS, 1% P/S, 1% L-glutamine and 1% Hepes (GE Healthcare). Lymph node cells (LNCs) were isolated as previously described^72^. Briefly, lymph node samples were washed twice with DPBS, excess adipose tissue and blood vessels removed, and remaining tissue passed through a 70 µm nylon cell strainer. The filtrate was suspended in 20 mL of LM. Debris was allowed to settle and the supernatant was centrifuged at 300 *g* for 15 min. The cell pellet was re-suspended in media and washed again. The resultant pellet was re-suspended and cells counted using Trypan blue dye exclusion. Cell were cryopreserved at 1 × 10^7^ mL^−1^ in CellBanker 2 medium in a freezing container (Mr Frosty, Thermo Fisher Scientific) to chill at −1 °C min^−1^ to −80 °C and then transferred to liquid nitrogen storage after 24 h.

### Dog leukocyte antigen genotyping

Dog leukocyte antigen (DLA)-88 (encoding MHC I) was genotyped for each dog using the GenElute™ Mammalian Total RNA Miniprep Kit (Sigma-Aldrich) to extract RNA from CDCs. RNA was reverse transcribed using ImProm-II™ Reverse Transcription System (Promega), and DLA-88 genes amplified by polymerase chain reaction (PCR) using MyTaq DNA Polymerase (Bioline) with the forward primer: 5′-CGGAGATGGAGGTGGTGA-3′ and reverse: 5′-GTGGCGGGTCACACG-3′ as previously described for amplification of DLA-88^73^.

DLA-DRB1 (encoding MHC II) was genotyped from genomic DNA (gDNA), extracted from CDCs using a commercial kit (GenElute™ Mammalian Genomic DNA Miniprep Kit; Sigma-Aldrich). The gene was amplified using the M13-adapted forward primer: 5′-TGTAAAACGACGGCCAGTCTCACTGGCCCGGCCTGTCTC-3′ and reverse: 5′-CACCTCGCCGCTGAACGTG-3′. PCR products were purified using GenElute™ PCR Clean-up Kit (Sigma-Aldrich) and purified amplicons sent for sequencing (Source Bioscience). Obtained sequences were analysed and MHC alleles assigned using the SBT Engine software package version 3.6.1 (GenDx, Genome Diagnostics B.V.).

### Mixed lymphocyte reaction

A mixed lymphocyte reaction (MLR) was used to assess the proliferative response of responder LNCs to allogeneic CDCs. LNCs were rapidly thawed at 37 °C, pelleted (300 *g* for 15 min) and re-suspended in LM at a density of 2 × 10^6^ cells mL^−1^. Cryopreserved CDCs were first cultured for 3–5 days as described for fresh cells. These were then detached from tissue culture vessels, counted, adjusted to the concentrations as required and seeded into assays. MLRs were run for 5 days, and wells pulsed with 1 µCi [^3^H]-thymidine (Perkin-Elmer) for the final 18 h of culture. Plates were harvested onto glass-fibre mats (Perkin-Elmer) using a Tomtec 96 cell harvester (Wallac). [^3^H]-thymidine incorporation was quantified using a MicroBeta Trilux liquid scintillation counter (Perkin Elmer).

To determine the highest proliferative response, 2.2 × 10^4^ CDCs in 100 µL of media were added to a round-bottom 96-well plate (Thermo Fisher) and serial 10-fold diluted. LNCs (2 × 10^5^ in 100 µL) were added, resulting in a CDC:LNC ratio ranging from 1:10 to 1:10^7^. The ratio giving the highest proliferative response was then used for subsequent MLRs. Concanavalin A (ConA, Sigma Aldrich) was added at 5 µg mL^−1^ where indicated. To assess the impact of CDCs on activated LNCs, LNCs were incubated with ConA for 24 h after which time CDCs were added. Where indicated, PGE_2_ (R&D Systems) was added to LNC cultures at a concentration of 1 µM and the selective EP4 antagonist L-161982 (Sigma Aldrich) added at a concentration of 10 µM. In some experiments, indomethacin (20 µM; Sigma Aldrich) was added to wells containing 2 × 10^5^ LNCs and 2 × 10^4^ CDCs to assess the role of prostaglandin synthesis. To investigate the role of proinflammatory cytokines in PGE_2_ production by CDCs, cells were cultured in the presence of recombinant canine interferon gamma (IFN-γ; 100 ng mL^−1^) and/or recombinant canine tumour necrosis factor alpha (TNF-α; 50 ng mL^−1^), and supernatants removed after 48 h for analysis of PGE_2_ by ELISA as detailed below. All experiments were run in triplicate using cells generated from at least 3 different dogs.

### Impact of cell-cell contact and soluble mediators on lymphocyte activation

To investigate whether a potential effect on cell death and lymphocytes activation of CDCs with allogeneic LNCs requires cell-cell contact and/or the presence of soluble mediators, two different co-cultures systems were established in 24-well plates.

In the first system, CDCs were detached from tissue culture vessels, pelleted (400 *g* for 7 min), re-suspended in CEM and counted. Next, 5 × 10^5^ CDCs were seeded per well of a 24-well plate. These were allowed to adhere for 24 h, after which non-attached cells were removed by washing twice with 1 mL DPBS. LNCs were then thawed rapidly at 37 °C, re-suspended in LM, centrifuged at 300 *g* for 15 min and 1 × 10^6^ cells added per well. To explore the effect of CDCs on activated LNCs, LNCs were also cultured with CDCs in the presence of ConA.

To assess the impact of soluble mediators, a transwell system was employed. 5 × 10^5^ CDCs were placed in the insert of a 6.5 mm transwell support (Thermo Fisher Scientific). Cells were allowed to adhere for 24 h, after which time the media was changed and 1 × 10^6^ LNCs added to the lower chamber in the presence of ConA. All experiments were run for 4 days then harvested for FACS analysis or cells re-plated in a 96-well plate and pulsed with [^3^H]-thymidine for 18 h as described for the MLRs.

### Antibody staining and flow cytometry

Cryopreserved CDCs were thawed and cultured for 3–5 days as above prior to antibody staining. CDCs then were washed twice with DPBS, detached from culture vessels (0.25% trypsin for 5 min), pelleted (400 *g* for 7 min) and re-suspended in chilled (4 °C) FACS buffer (FACSFlow; BD Bioscience) at 3 × 10^6^ mL^−1^. Aliquots (100 µL) were transferred to FACS tubes (Thermo Fisher Scientific). For cells in co-culture the supernatant was first removed and wells washed twice with 1 mL DPBS. Adherent cells were detached by a 5 min incubation with 0.25% trypsin and cells along with supernatant and washing DPBS from one well pooled and pelleted at 400 *g* for 7 min. Cells were re-suspended at a concentration of 1–3 × 10^6^ mL^−1^ in 100 µL of buffer for antibody staining in FACS tubes (Thermo Fisher Scientific). Cells were then incubated with monoclonal antibodies (mAb) for 30 min at 4 °C protected from light. Tubes were centrifuged at 400 *g* for 5 min and cell pellet re-suspended in 1 mL of chilled buffer for acquisition, or 100 µL of buffer for secondary mAb staining. Secondary staining followed the same procedure as for primary mAbs. The primary mAbs used were: mouse anti-canine CD25 (Bio-Rad, FITC, clone P4A10, dilution 1:10), mouse anti-bovine MHC I (Kingfisher Biotech, unconjugated, clone H58A, 1:10) and rat anti-canine MHC II (eBioscience, APC, clone YKIX334.2, 1:10). Secondary mAb for MHC I was rat anti-mouse IgG2a (Biolegend, PE, clone RMG2a-62, 1:10). Isotope controls were mouse IgG1 (Bio-Rad, 1:10) and rat IgG2a (eBioscience, 1:10). Prior to acquisition, co-culture cells were incubated for 15 min at room temperature with 10 µL of propidium iodide (PI, Invitrogen, 10 µg mL^−1^). Additionally, co-cultures or CDCs alone were assessed for apoptosis using the Annexin V Apoptosis Detection Kit according to the manufactures instructions (Thermo Fisher).

Stained samples were acquired on a BD FACS Calibur flow cytometer using CellQuest Pro software (both BD Bioscience). Prior to acquisition the cytometer was calibrated using CaliBRITE 4 colour FACS Comp beads (BD Bioscience). The forward and side scatter parameters were adjusted in order to centre the cell population on the scatter plot (for CDCs) or to enable visualisation of both LNCs and CDCs. Fluorescence intensity was adjusted to set the unlabelled cells within 10^0^–10^1^ on the log scale axis. Cells were acquired with an event count set to 1 × 10^4^ of the gated events (excluding debris). Data was analysed using FlowJo software (FlowJo, LLC).

### ELISA for canine TGF-β1 and PGE_2_

Supernatants (100 µL) were removed from MLR and transwell experiments immediately prior to the addition of [^3^H]-thymidine and stored at −20 °C for cytokine analysis. To determine the total amount of TGF-β1 and PGE_2_ in cell free supernatants, commercial ELISA kits specific for canine TGF-β1 and PGE_2_ was used according to the manufacturers instructions (Quantikine ELISA Kit, R&D Systems).

### Statistical analysis

The SPSS software package (IBM, version 23 for Mac) was used for statistical analysis. Data was assessed for normality using histogram analysis. All data are presented as the mean ± SEM of three pooled data sets from three sets of dogs, with samples run at least in triplicate. Comparisons between two independent samples were performed using Student’s two-tailed T-test and between three or more groups using one-way ANOVA with post-hoc Tukey analysis. A p value of <0.05 was considered significant.

## Electronic supplementary material

Supplementary Figures S1, S2 and S3

## Data Availability

The datasets generated during this study are archived on Institutional secure servers and may be available on reasonable request.
