# Supplementary material for: Cardiosphere-derived cells suppress allogeneic lymphocytes by production of PGE2 acting via the EP4 receptor
Source: Sci Rep. 2018 Sep 6;8:13351. doi: 10.1038/s41598-018-31569-1 (PMC6127326; doi:10.1038/s41598-018-31569-1)
Supplement: Supplementary file 1 — Supplementary Figures S1, S2 and S3 [file 41598_2018_31569_MOESM1_ESM.pdf]

Cardiosphere-derived cells suppress allogeneic lymphocytes by production of PGE_2 acting via the EP4 receptor

Luke C. Dutton[1, *], Jayesh Dudhia[1], Brian Catchpole[2], Hannah Hodgkiss-Geere[3], Dirk Werling[2, 4], and David J. Connolly[1, 4]

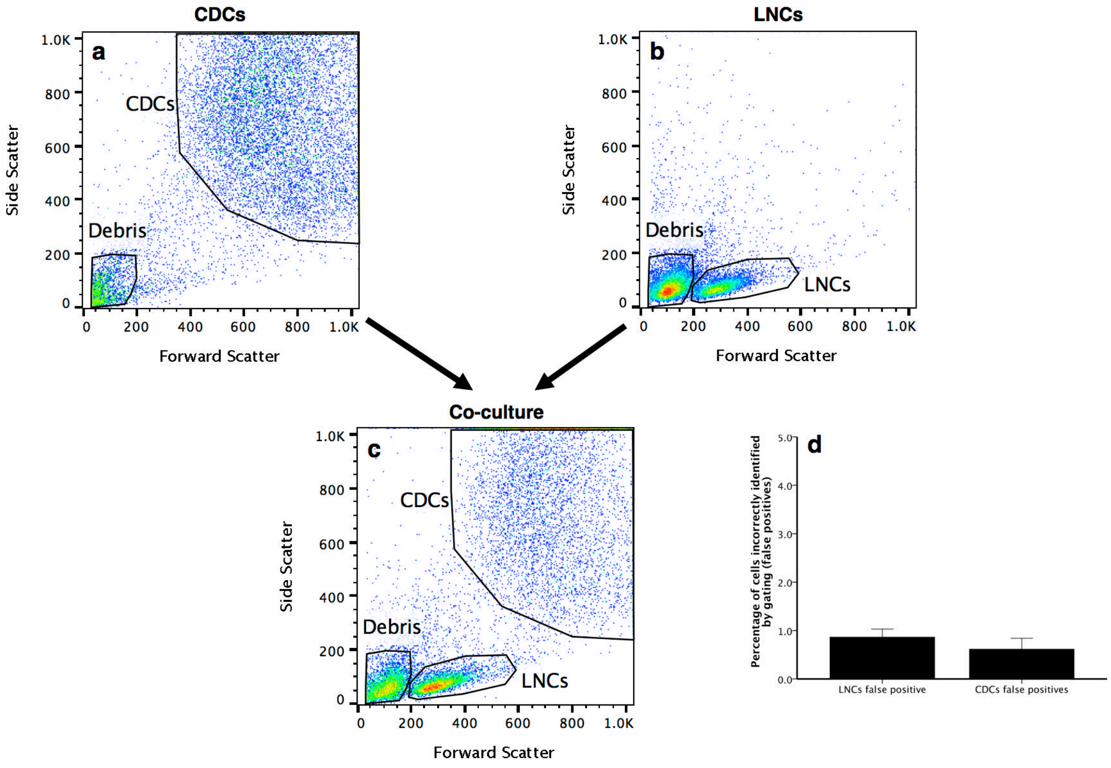

Supplementary Figure S1: Gating of flow cytometry experiments for analysis of cardiosphere-derived cells (CDCs) and lymph node cells (LNCs) separately. Running forward and side scatter parameters for CDCs (a) and LNCS (b) individually allowed gates to be constructed around the known populations. Debris could then also be excluded. Unique side and forward scatter properties of the cells allowed identification in the co-culture system (c). The estimated false positive rate (i.e. the number of LNCs gated when only CDCs are present; LNCs false positives, and the number of CDCs gated when only LNCs are present; CDC false positives) is <1% (n = 3 dogs, mean ± SEM, d)

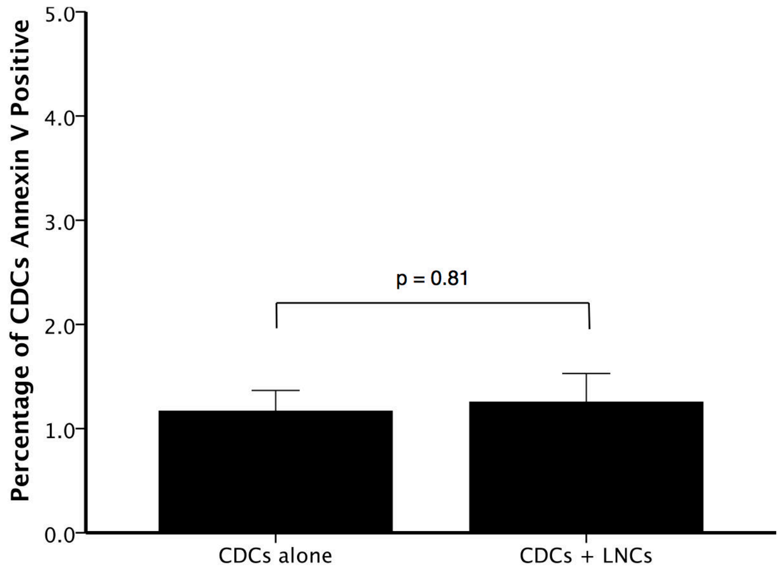

Supplementary Figure S2: Annexin V staining indicated that CDCs did not undergo apoptosis when co-cultured with LNCs. The percentage of Annexin V positive CDCs was similar when CDCs are cultured alone versus when cultured with LNCs (n = 3 dogs, p = 0.81). Bars represent mean ± SEM.

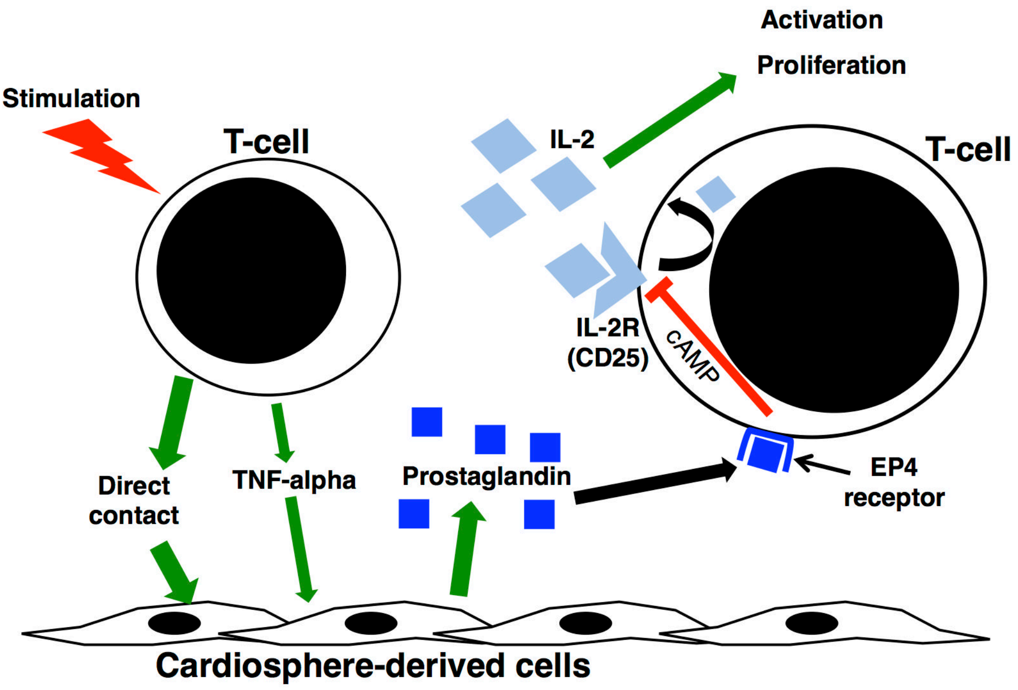

Supplementary Figure S3: Graphical representation of the mechanism governing cardiosphere-derived cell (CDC) immunosuppression. Lymphocytes (designated T-cells here) are first activated by an external stimulus, causing release of pro-inflammatory cytokines such as TNF-α. Activated lymphocytes also act via direct contact on CDCs. These cause the CDCs to produce and secrete prostaglandin, specifically PGE_2, into the local environment. Direct cell-cell contact appears to be a more potent stimulator of PGE_2 release from CDCs versus inflammatory cytokines. PGE_2 then binds to receptor subtype EP4 on the lymphocytes, triggering an inhibitory pathway mediated via increased cytosolic cAMP. This leads to down-regulation of the interleukin-2 receptor (IL-2R) and IL-2 release from lymphocytes. This then inhibits IL-2 mediated lymphocyte activation and expansion, thus leading to suppression of lymphocyte proliferation.
